# Multi-task learning-based feature selection and classification models for glioblastoma and solitary brain metastases

**DOI:** 10.3389/fonc.2022.1000471

**Published:** 2022-09-21

**Authors:** Ya Huang, Shan Huang, Zhiyong Liu

**Affiliations:** ^1^Key Laboratory of Computing and Stochastic Mathematics, School of Mathematics and Statistics, Hunan Normal University, Changsha, China; ^2^Department of Intensive Care, Xiangya Hospital, Central South University, Changsha, China; ^3^National Clinical Research Center for Geriatric Disorders, Xiangya Hospital, Central South University, Changsha, China

**Keywords:** solitary brain metastases, glioblastoma, multi-task learning, feature selection, classification, logistic regression

## Abstract

**Purpose:**

To investigate the diagnostic performance of feature selection *via* a multi-task learning model in distinguishing primary glioblastoma from solitary brain metastases.

**Method:**

The study involved 187 patients diagnosed at Xiangya Hospital, Yunnan Provincial Cancer Hospital, and Southern Cancer Hospital between January 2010 and December 2018. Radiomic features were extracted from conventional magnetic resonance imaging including T1-weighted, T2-weighted, and contrast-enhanced T1-weighted sequences. We proposed a new multi-task learning model using these three sequences as three tasks. Multi-series fusion was performed to complement the information from different dimensions in order to enhance model robustness. Logical loss was used in the model as the data-fitting item, and the feature weights were expressed in the logical loss space as the sum of shared weights and private weights to select the common features of each task and the characteristics having an essential impact on a single task. A diagnostic model was constructed as a feature selection method as well as a classification method. We calculated accuracy, recall, precision, and area under the curve (AUC) and compared the performance of our new multi-task model with traditional diagnostic model performance.

**Results:**

A diagnostic model combining the support vector machine algorithm as a classification algorithm and our model as a feature selection method had an average AUC of 0.993 in the training set, with AUC, accuracy, precision, and recall rates respectively of 0.992, 0.920, 0.969, and 0.871 in the test set. The diagnostic model built on our multi-task model alone, in the training set, had an average AUC of 0.987, and in the test set, the AUC, accuracy, precision, and recall rates were 0.984, 0.895, 0.954, and 0.838.

**Conclusion:**

It is feasible to implement the multi-task learning model developed in our study using logistic regression to differentiate between glioblastoma and solitary brain metastases.

## 1 Introduction

Brain tumors, also known as intracranial tumors, are a growth or mass of abnormal cells or tissue in the brain (1). Brain tumors are generally subdivided into two main types: primary or secondary (metastatic) (2). Glioblastoma (GBM) is a typical malignant primary brain tumor that affects an average of 3 out of 100,000 people (3). Solitary brain metastases (SBM) are secondary malignant brain tumors, which are more common than GBM. The incidence rate of SBM is approximately 7 to 14 per 100,000 people (3, 4). As the standard treatment course for GBM is aggressive trimodality therapy compared to surgery or radiosurgery for SBM, it is of great clinical importance to accurately and rapidly distinguish between these two types of brain malignancies as rapidly as possible. As the main diagnostic tool for brain tumors (5), magnetic resonance imaging (MRI) methods create clear and detailed three-dimensional images of brain and tumor anatomy. However, for patients with SBM and GBM, their MR images both show ring enhancement, intra-tumor necrosis, and per femoral T2 high signal (6, 7), which poses a challenge for the accurate differentiation between GBM and SBM.

Radiomics (8–12), the application of advanced image feature analysis algorithms, can be used to capture intra-tumoral heterogeneity in a non-invasive manner. Numerous studies have applied radiomics to tumor classification. Austin et al. used a filtered histogram texture analysis-based imaging historic approach to identify high-grade and low-grade gliomas (13), where the AUC on the test set reached 0.90. However, the data of their study were highly unbalance in the number of high-grade and low-grade gliomas. Among the three feature selection methods, packing, filtering, and embedding, the embedding method can obtain a higher computational efficiency and classification performance than the filtering (14, 15) and packing (16) methods (17). Therefore, the embedding method has received increasing attention recently. Qian et al. used the feature selection method of least absolute shrinkage and selection operator (Lasso) combined with a support vector machine(SVM) classifier, to obtain an AUC of 0.90 in their test set (18). Cho et al. used a machine learning approach to classify gliomas based on radiomics (19), which ultimately selected five significant features with an average AUC of 0.903 on their test set. Artzi et al. found that the SVM approach had the best results for classifying between GBM and SBM subtypes (20), with an AUC of 0.96.

Liu et al. combined handcrafted radiomics and deep learning-based radiomics and used a random forest algorithm for feature selection and classification (21), the AUC reached 0.97 for single contrast-enhanced T1-weighted(T1C) MRI sequence. Because tumor sites behave differently under different sequences of MRI, patients usually have multiple series of imaging data acquired to accurately determine the tumor location, size, and additional information during the treatment. Different sequences provide different information, and multiple sequence fusion can complement information from different dimensions, thus enhancing the robustness of a model. As such, we introduced a multi-task learning model (22, 23) to fuse T1, T1C, and T2 sequence information to develop a robust prediction model to aid in clinical diagnosis.

Nowadays, most studies on multi-task-based feature selection focus on different canonical terms. The features are selected by constraints of different paradigms, such as the commonly used *ℓ*_1,1_ (24), *ℓ*_2,1_ (25) paradigms, etc. (17, 26). In the present report, we have improved the data-fitting term in the multi-task learning model so that the model can be used not only for feature selection but also for classification functions to ultimately achieve a higher accuracy than traditional diagnostic models.

The present work proposes a new feature selection and classification model based on the multi-task learning model, which treats the 1106 features extracted from each sample in each sequence (T1, T2, T1C) as a task. It uses the logical loss function as a data-fitting term to ensure the feasibility of classifying GBM and SBM. Taking *ℓ*_1,1_, *ℓ*_1_ as regular terms to ensure the sparseness of feature selection. At the same time, private weights are introduced based on a common weight to make full use of the relevant similarities and differences between each task. The result is a 3-task feature selection classification model. In this study, we mainly utilized the alternating iterative method and the fast-iterative shrinkage threshold algorithm based on the backtracking method to solve equations. The experimental results demonstrate that our model, whether as a feature selection model combined with SVM classification methods to form a diagnostic model or as a standalone diagnostic model, successfully integrated multiple sequence information to provide a robust predictive model for clinical diagnosis while also the diagnostic model consisting of one model improves the efficiency of our tumor classification.

## 2 Materials and methods

### 2.1 Data acquisition

The data used in this study were obtained from 120 patients with SBM and 67 patients with GBM admitted to the Xiangya Hospital, Yunnan Cancer Hospital and Southern Cancer Hospital between January 1, 2010 and December 31, 2018. All patients were histologically diagnosed according to the tumor grading guidelines published by the World Health Organization in 2021. This retrospective analysis of data from MR images was approved by the institutional review board, and the requirement of informed consent was waived.

MRI on all patients was performed by the hospital radiology department using 3.0-T systems. Each patient had T1, T2, and T1C MR image series performed. High-quality MR images were obtained using the following protocols:

- Axial T1: layer thickness =5 mm, layer spacing =1.5 mm, matrix =320×256, and field of view (FOV)=24×24 cm.
- Axial T2: layer thickness =5 mm, layer spacing =1.5 mm, matrix =384×384, and FOV =24×24 cm.
- Axial T1C: layer thickness =5 mm, layer spacing =1.5 mm, matrix =320×256, and FOV =24×24 cm.

MR image data of the patient for the present study can be found in the reference (21).

### 2.2 Data preprocessing

**Figure 1:**
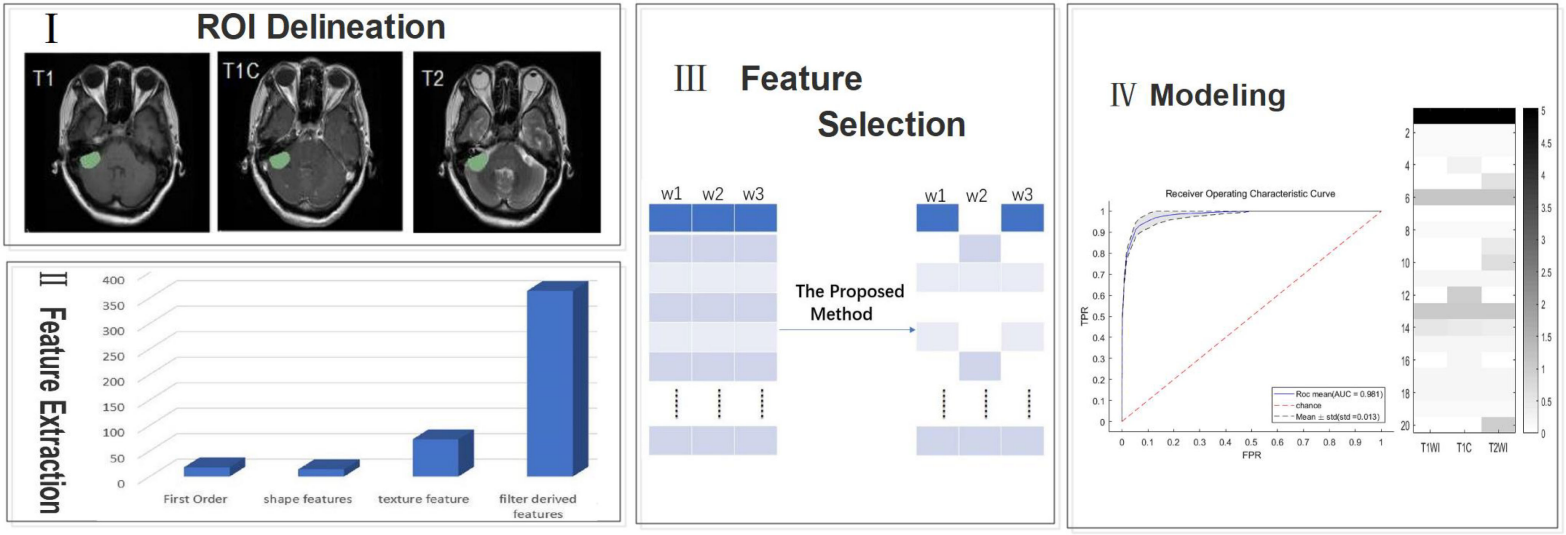
A generic framework for creating classification models using radiological features. Steps include ROI(region of interest) delineation, feature extraction, feature selection, and modeling.

The overall workflow of the current study is shown in **Figure 1** with a description of each involved step.

#### 2.2.1 Delineation of the region of interest (ROI)

We preprocessed each image by noise reduction, offset field correction, and strict intra-target alignment using the public software package FSL v6.0.4. All images were evaluated independently by two neuroradiologists who have between 5-10 years of experience. ROIs of the entire tumor on T1, T2, and T1C images were created manually using the ITK-SNAP software layer by layer around the enhanced tumor layer (27); areas of macroscopic necrosis, cystic degeneration, and edema were avoided. A third senior neuroradiologist with 15 years of experience reexamined the images and made a final diagnosis when there was a conflict between the two original neuroradiologists (21).

#### 2.2.2 Data normalization

Differences in instrumentation and imaging parameters, tumor sites of patients, and other factors can lead to significant differences in MR images. These differences will result in significant issues for imaging histology analysis. Therefore, we performed MIL(modality mismatch, intensity distribution variance, and layer-spacing differences) normalization for all MR images (28). First, we used B-sample interpolation for body mode matching for all patient MR images to obtain a total of 120 SBM samples and 67 GBM samples, Second, we set the interlayer gap of all MR images to 1 mm. Third, we applied MIL data normalization to make the intensity of MR image distribution consistent.

#### 2.2.3 Feature extraction

We used the platform PyRadiomics (http://www.radiomics.io/pyradiomics.html) to perform feature extraction on all MIL normalized data. 1106 features were extracted from each MRI series. With each patient undergoing three different sequences of MRI, we extracted a total of 3318 features per patient. The extracted features are shown in **Table 1**.

**Table 1:** Extract the specific content of radiomics features.

Original image		Derived image	
feature	number	name	number
Shape-based	14	LoG	273
First Order	18	Wavelet	364
Gray Level Co-occurrence Matrix	22	Square	91
Gray Level Run Length Matrix	16	Square Root	91
Gray Level Size Zone Matrix	16	Logarithmic	91
Gray Level Dependence Matrix	14	Exponential	91
Neighboring Gray Tone Difference Matrix	5	–	–

To account for the large difference in the number of samples between the two tumor groups, we used the Synthetic Minority Over-sampling Technique (SMOTE) (29) to oversample the GBM group in order to generate the same number of samples as the SBM group. In total, we obtained 120 SBM samples and 120 GBM samples. Finally, we randomly selected 24 SBM samples and 24 GBM samples as the test set, and the remaining 96 SBM samples and 96 GBM samples as the training set.

### 2.3 Feature selection and classification

#### 2.3.1 Proposed model

We introduce the logical loss function into the multi-task learning model to obtain the data fitting term as


(1) $$L(X,y,W)=\sum_{m=1}^{M}\sum_{j=1}^{N}(-y_{i}^{m}X_{i}^{m}w^{m}+\ln\;(1+\exp\;(X_{i}^{m}w^{m}))$$

where the number of samples for each task is *N* and $y_{j}^{m}\in \{+1,-1\}$ denotes the class of samples. When y=−1 , the patient has GBM. y=+1 means the patient has SBM.

To obtain the characteristics unique to a single task, we introduced a personal value of *b* based on the expected weight of *s* and finally derived our new model


(2) $$\underset{s,B}{\min}\;\sum_{m=1}^{M}\sum_{i=1}^{N}\frac{1}{N}(-y_{i}X_{i}^{m}(s+b^{m})+\ln\;(1+\exp\;(X_{i}^{m}(s+b^{m}))))+\lambda_{s}{║s║}_{1}+\lambda_{b}{║B║}_{1,1}$$

Where $y_{i}$ is the label of the $i^{th}$ sample. *M* is the number of tasks where *M=3* as described in the text. We assume that the number of samples for all tasks is *N*. $X_{i}^{m}\in {\mathbb{R}}^{1\times (d+1)}$ is the $i^{th}$ sample of the $\;m^{th}$ task, i.e., the $i^{th}$ row of the matrix $X^{m}$ . Depending on the nature of logistic regression, we populate the last column of $X^{m}$ with an N-dimensional 1 vector. $B=[b^{1},b^{2},\cdots ,b^{M}]\in {\mathbb{R}}^{(d+1)\times M}$ , then $b^{m}$ is the $m^{th}$ column of matrix *B*, ${\left||s|\right|}_{1}=\overset{d+1}{\underset{i=1}{\sum }}|s_{i}|$ and ${\left||B|\right|}_{1,1}=\overset{\left(d+1\right)}{\underset{i}{\sum }}\overset{M}{\underset{m}{\sum }}|b_{i}^{m}|$ are the regularization terms where *s* and bmare are the shared and private weights, respectively. $\lambda_{s},\lambda_{b}$ are the two regularization parameters, and let $\lambda_{s}<M\lambda_{b}$ .

#### 2.3.2 Model solving

We rewrote (2) in the following form


(3) $$\underset{s,B}{\min}\sum_{m=1}^{M}\sum_{i=1}^{N}\frac{1}{N}(-y_{i}X_{i}^{m}(s+b^{m})+\ln\;(1+\exp\;(X_{i}^{m}(s+b^{m}))))+{\text{λ}}_{s}\sum_{i}^{p+1}\left|s_{i}\right|+{\text{λ}}_{b}\sum_{i}^{d+1}\sum_{m}^{M}\left|b_{i}^{m}\right|$$

We considered the matrix *B* as a constant matrix, with a minimization of the variable *s*


(4) $$\underset{s}{\min}\;\sum_{m=1}^{M}\sum_{i=1}^{N}\frac{1}{N}(-y_{i}X_{i}^{m}(s+b^{m})+\ln\;(1+\exp\;(X_{i}^{m}(s+b^{m}))))+\lambda_{s}\sum_{i}^{p+1}|s_{i}|$$

Similarly, fixing the vector *s* and considering it as a constant vector, then minimizing *B* is equivalent to minimizing the following problem for any *m*


(5) $$\underset{b^{m}}{\min}\;\sum_{i=1}^{N}\frac{1}{N}(-y_{i}X_{i}^{m}(s+b^{m})+\ln\;(1+\exp\;(X_{i}^{m}(s+b^{m}))))+\lambda_{b}\sum_{i}^{d+1}|b_{i}^{m}|$$

It is further shown that the computation of $b^{m}$ is only relevant for a single task.

To select the final feature, equation (4) and (5) must be solved. Because of the non-differentiation of the $\ell_{1}$ norm, we used a fast-iterative shrinkage threshold algorithm to solve the above two subproblems in the course of our study (30). Both equations were solved in a similar manner, but the calculation in the model (5) involved only the $m^{th}$ task, independent of the other tasks, while solving model (4) required the participation of all tasks.

#### 2.3.3 Model analysis

**Figure 2:**
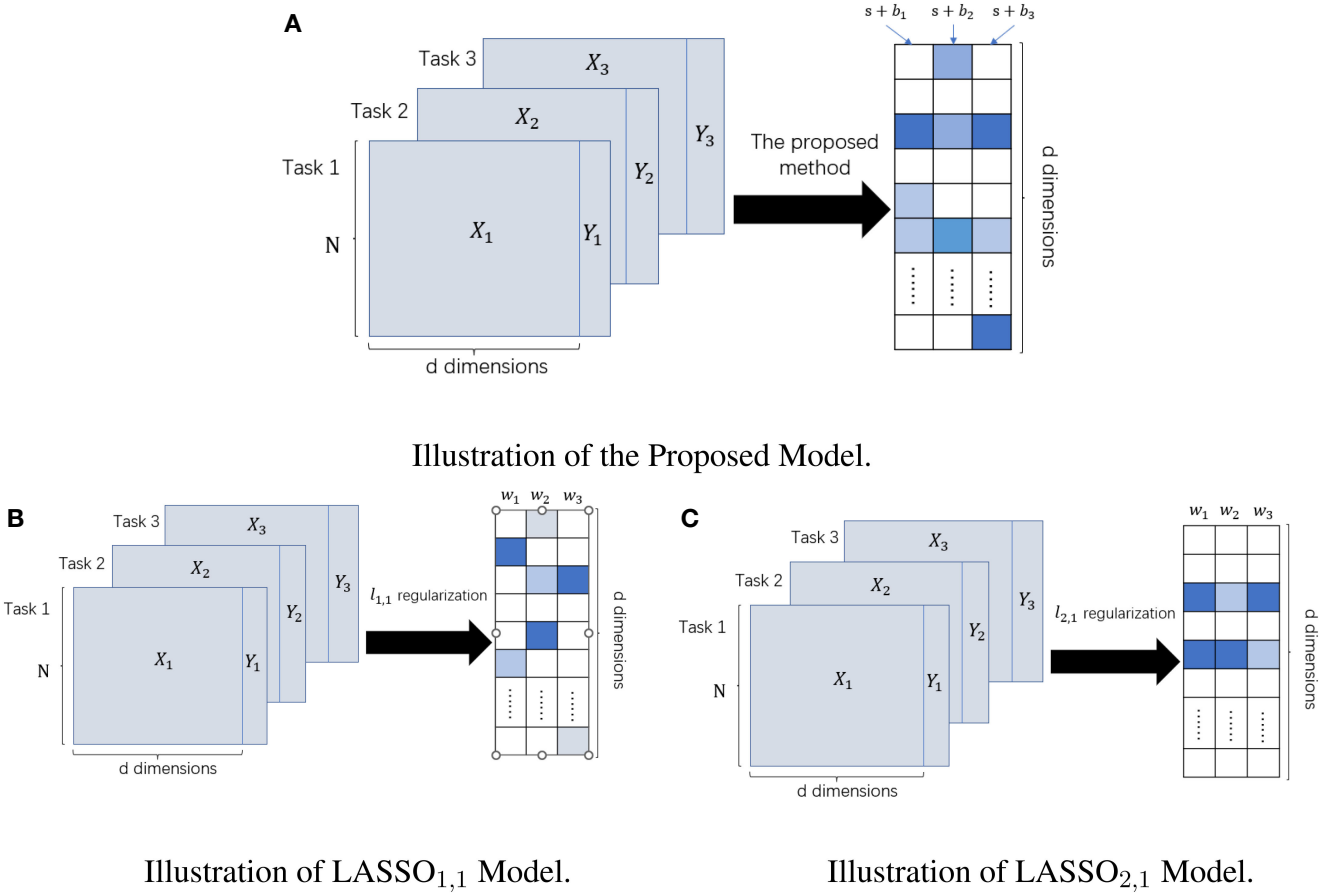
The difference between LASSO_1,1_ Model, LASSO_2,1_ Model, and Our Proposed Model. The left panel represents the input datasets; the right panel represents the learned weight matrix. **(A)** Schematic of our method for feature selection. **(B)** Schematic diagram of feature selection based on multi-task Lasso model under ℓ_1,1_ regularization. **(C)** Schematic diagram of feature selection based on multi-task Lasso model under ℓ_2,1_ regularization.

We represent the weights solved by the model in the form of a sum of shared weights *s* and private weights $b^{m}$ . For the computation of s, all tasks need to be involved, and when $s_{i}\neq 0$ , it is assumed that all tasks pick the $i^{th}$ feature. However, the computation of $b^{m}$ requires data from only the $m^{th}$ task, and $b^{m}\neq 0$ indicates the $i^{th}$ feature is important for the $m^{th}$ task but may not be important for other tasks. Finally, we denoted the features selected by the mth task by $\left(s+b^{m}\right)$ . The $\ell_{1}$ and $\ell_{1,1}$ regularization forced both weights to be sparse, thus satisfying the “feature selection” requirement. The feature selection methods with $\ell_{1,1}$ norm as the regular term or $\ell_{2,1}$ norm as the regular term are two classical methods in the sparse embedding method (31). The multi-task Lasso model based on $\ell_{1,1}$ regularization had an entirely separable form. Each task can separately compute its own weight without being influenced by other tasks, so the sparse multi-task Lasso model is equivalent to the single-task Lasso model. The single difference is that all tasks of the sparse multi-task Lasso method use the same regularization parameter. In contrast, the regularization parameter of the single-task Lasso model can be determined by each task individually. The multi-task Lasso model based on the $\ell_{2,1}$ regularization makes the features selected by different tasks almost the same, which reasonably exploits the correlation between different tasks but loses the specificity between different tasks. In contrast, our model not only makes full use of the correlation between different tasks but also highlights the specificity between different tasks and fuses the information of multiple sequences, thus providing a more robust prediction model for clinical diagnosis. Their differences are given visually in **Figure 2**.

Accurate preoperative diagnosis can be effective in formulating accurate and personalized treatment for patients, especially when MR images of SBM and GBM are extremely similar. In the current study, the proposed multi-task learning based on the logistic loss function can be used not only for feature selection but also for tumor classification tasks.

In contrast, the Lasso model based on mean square loss can only be used as a feature selection algorithm and cannot perform the subsequent classification task independently. In contrast, our model can use probabilities to account for the classification, for example, for any sample $X_{i}^{m}\in {\mathbb{R}}^{1\times (d+1)}$ , the probability that the sample is classified as label 1 is


(6) $$p(y_{i}=1|X_{i}^{m})=\frac{1}{1+e^{-X_{i}^{m}(s+b^{m})}}$$

Next, the model can perform the tumor classification task independently by simply picking the appropriate threshold value. Its calculation process will be described in the section of experimental procedure.

#### 2.3.4 Experimental procedure

**Figure 3:**
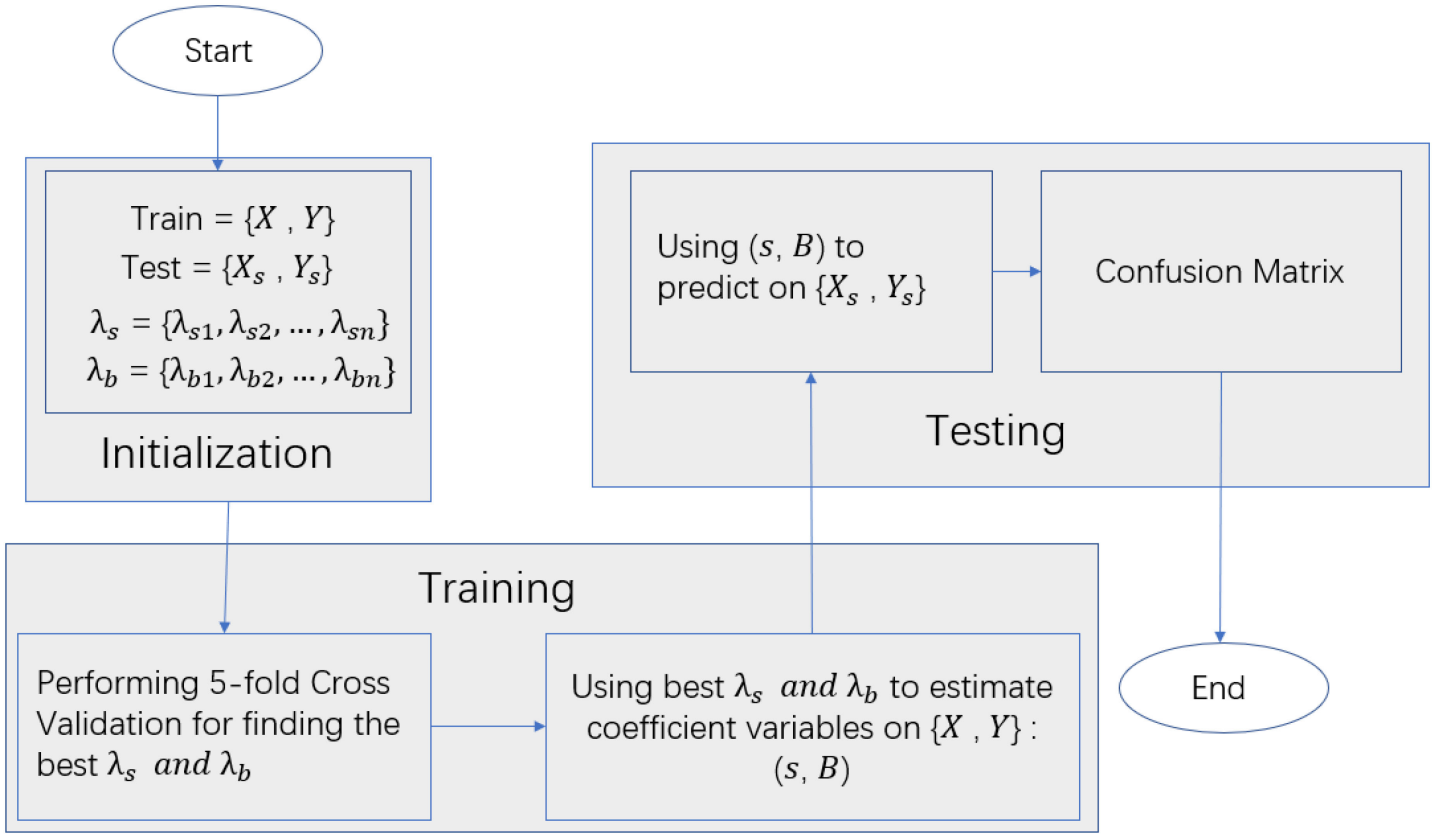
Experimental flowchart.

This experiment was performed using MATLAB 9.11. The features of each sample and the labels are used as input, and the optimal penalty parameters $\lambda_{s}$ and $\lambda_{b}$ are obtained on the training set using 5-fold cross-validation. The ratio $\frac{\lambda_{s}}{\lambda_{b}}$ of the two penalty parameters is always kept as the following six values: 1.25, 1.5, 1.75, 2, 2.25, 2.5. Moreover, the mean AUC values under the optimal penalty parameters are recorded. Then, the data were computed and trained using the alternating miniaturization algorithm and the fast-iterative shrinkage threshold algorithm. Note that during the fast-iterative shrinkage threshold algorithm solution, our parameters $L_{0}=1,\eta =1.1$ . To ensure the confidence of the results, we repeatedly performed 5-fold cross-validation 10 times. Finally, the model"s performance was evaluated by average accuracy, average recall, average precision, and average AUC(The flow chart of our model solution is given in **Figure 3**).

## 3 Results

Additional analyses were conducted to compare our model with four diagnostic models based on single-task and multi-task learning. The single-task-based models are the diagnostic model with Lasso model for feature selection and SVM for classification (Lasso+SVM); the diagnostic model with Lasso model for feature selection and logistic regression (LR) algorithm for classification (Lasso+LR); the logistic regression as loss function and $\ell_{1}$ norm constraint of “LR_1_” model for feature selection and SVM for classification (LR_1_+SVM); and a diagnostic model with LR_1_ model for feature selection and classification (LR_1_).

The models based on multi-task learning are the diagnostic model with the Lasso multi-task model with $\ell_{1,1}$ norm as the regular term for feature selection and the SVM method for classification(Lasso_1,1_+SVM); the diagnostic model using the Lasso_1,1_ multi-task model for feature selection and logistic regression(LR) for classification(Lasso_1,1_+LR); diagnostic model with feature selection using the Lasso multi-task model with $\ell_{2,1}$ norm as the regular term and classification by SVM method(Lasso_2,1_+SVM); diagnostic model with feature selection using Lasso_2,1_ multi-task model and classification by LR method(Lasso_2,1_+LR); our model as a diagnostic model with feature selection method and SVM method for classification(Ours+SVM) and our model as a diagnostic model(Ours).

**Table 2** shows the confusion matrices of 4 single-task models based on T1, T1C, and T2 sequences and 6 diagnostic models based on multi-task learning. The values in the table are taken as an average of the results of the 5-fold cross-validation 10 times. In the table, TP (True Positive) represents the number of GBM predicted as GBM, FN (False Negative) means the number of GBM predicted as SBM, FP (False Positive) means the number of SBM predicted as GBM, and TN (True Negative) represents the number of SBM predicted as SBM. From the experimental results, it can be seen that the value of TP or TN is significantly higher than the value of FN or FP. This shows that our model is meaningful and feasible.

**Table 2:** The mean of the confusion matrix of each model after 5-fold cross-validation 10 times.

		Test			
Group	Model	T P	F N	FP	TN
T1	Lasso+SVM	19.94	4.06	3.98	20.02
	Lasso+LR	19.78	4.22	4.54	19.46
	LR1+SVM	20.60	3.40	3.56	20.44
	LR_1_	20.58	3.42	3.86	20.14
T1C	Lasso+SVM	20.36	3.64	2.58	21.42
	Lasso+LR	19.64	4.32	3.40	20.60
	LR1+SVM	19.40	4.60	1.90	22.10
	LR_1_	19.18	4.82	1.98	22.02
T2	Lasso+SVM	20.50	3.50	3.38	20.62
	Lasso+LR	19.86	4.14	5.30	18.70
	LR1+SVM	20.60	3.40	2.56	21.44
	LR_1_	20.24	3.76	2.96	21.94
Multi-task	Lasso_1,1_+SVM	21.44	2.56	2.22	21.78
	Lasso_1,1_+LR	20.48	3.52	2.70	21.30
	Lasso_2,1_+SVM	20.16	3.84	1.64	22.36
	Lasso_2,1_+LR	20.32	3.68	2.64	21.36
	Ours+SVM	20.90	3.10	0.76	23.24
	Ours	20.12	3.88	1.16	22.84

**Figure 4:**
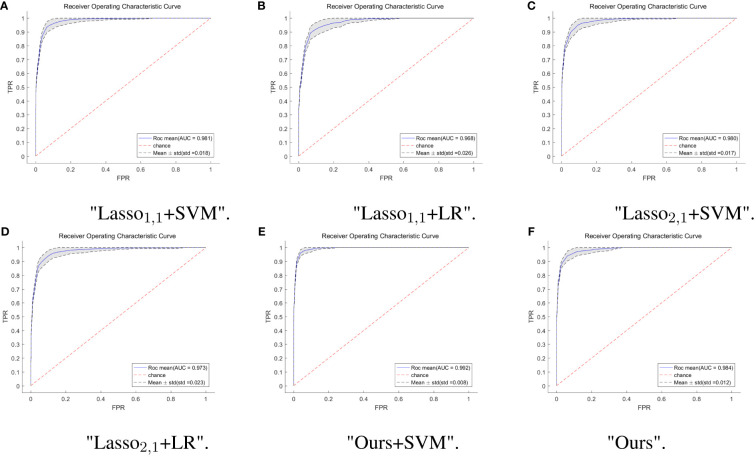
ROC curves of multi-task model in six. The horizontal coordinate indicates the false positive rate and the vertical coordinate indicates the true positive rate. **(A)** ROC curves for the Lasso_1,1_+SVM model, the blue solid line indicates the average ROC curve and the black dashed line indicates the mean ± std. **(B)** ROC curves for the Lasso_1,1_+LR model, the blue solid line indicates the average ROC curve and the black dashed line indicates the mean ± std. **(C)** ROC curves for the Lasso_2,1_+SVM model, the blue solid line indicates the average ROC curve and the black dashed line indicates the mean ± std. **(D)** ROC curves for the Lasso_2,1_+LR model, the blue solid line indicates the average ROC curve and the black dashed line indicates the mean ± std. **(E)** ROC curves for the Ours+SVM model, the blue solid line indicates the average ROC curve and the black dashed line indicates the mean ± std. **(F)** ROC curves for the Ours model, the blue solid line indicates the average ROC curve and the black dashed line indicates the mean ± std.

To further verify the feasibility of our model, the ROC (receiver operating characteristics) curves of our model are compared with those of four other multi-task models, and the values of the AUC and the standard deviation are also given. Usually, the closer to a value of 1 the AUC is, the better the model performance is; the smaller the standard deviation is, the more stable the model is. For the test dataset, our model combined with SVM classification, multi-task: Ours+SVM, had the most significant AUC of 0.992, however, our multi-task model alone had the second highest AUC of 0.984, with an AUC difference of 0.008 from the optimal model. The standard deviation (std) of the multi-task: Ours+SVM model is also the smallest among the six models, at 0.008, indicating that the model is the most stable. The std of our multi-task model alone was second only to the multi-task: Ours+SVM model. The results are shown in **Figure 4**.

**Figure 5:**
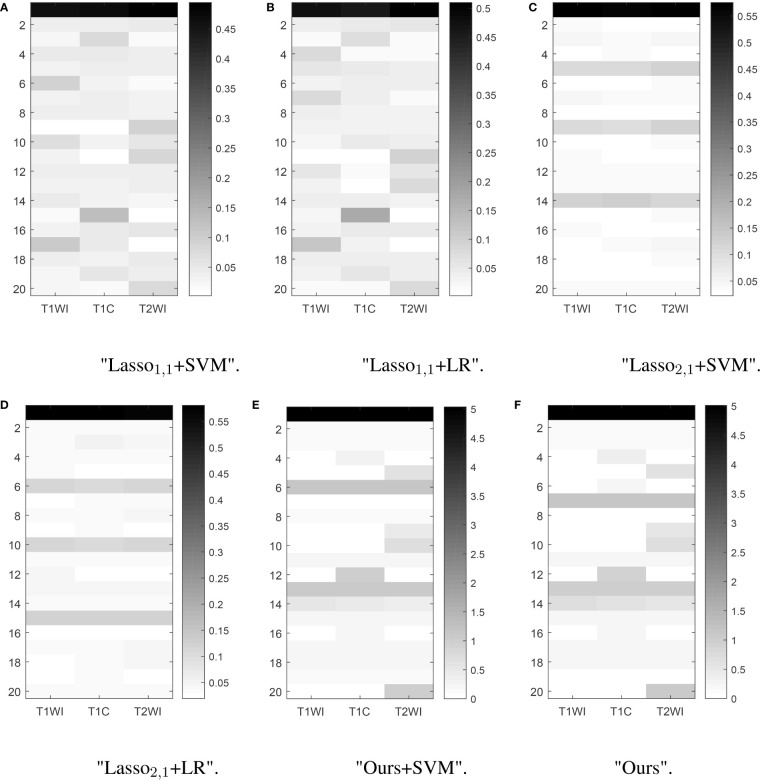
Learned weight matrix. The color bar in the right side indicates the values of matrix.The horizontal coordinates indicate the different tasks T1, T1C, and T2. The vertical coordinates indicate the number of features screened.

Feature selection can effectively reduce the dimensionality of the data, thereby reducing the amount of computation and improving the efficiency of problem-solving. **Figure 5** shows the distribution of features when selecting 20 features for six multitasking models. Our method not only selected the same features for the three sequences of T1, T1C, and T2, but also selected the characteristics that are unique to each sequence, which may have a large impact on only one of the sequences and not very much on the other sequences.

In brain tumor classification experiments, we compared the classification performance of the single-task-based and multi-task learning-based diagnostic models. In the single-task model experiments, we used four single-task feature selection models to classify the data of T1, T1C, and T2 sequences. Finally, we used 5-fold cross-validation method 10 times to obtain the average AUC on the training set and the average AUC, accuracy, precision, and recall on the test set. In the classification experiment based on multi-task learning, we treated the three sequences of T1, T1C, and T2 as 3 tasks, trained and tested them through 6 multi-task models, and obtained the above 4 evaluation indicators. The results were shown in **Table 3**.

**Table 3:** The values of various metrics for each method on the training and test sets.

		Train	Test				
Group	Model	AUC	AUC	Accuracy		Precision	Recall
T1	Lasso+SVM	0.936	0.933	0.832		0.851	0.830
	Lasso+LR	0.940	0.935	0.817		0.840	0.824
	LR_1_+SVM	**0.952**	**0.951**	**0.855**		**0.869**	**0.858**
	LR_1_	**0.951**	0.947	0.848		0.861	0.857
T1C	Lasso+SVM	**0.955**	0.953	**0.870**		0.901	**0.848**
	Lasso+LR	0.950	0.947	0.838		0.886	0.818
	LR_1_+SVM	**0.961**	**0.959**	0.864		**0.922**	0.818
	LR_1_	0.958	0.954	0.858		0.919	0.799
T2	Lasso+SVM	0.954	0.953	0.856		0.873	0.854
	Lasso+LR	0.947	0.942	0.803		0.828	0.854
	LR_1_+SVM	**0.973**	**0.969**	**0.876**		**0.828**	**0.858**
	LR_1_	0.968	0.967	0.860		0.892	0.843
Multi-task	Lasso_1,1_+SVM	0.984	0.981	0.900		0.918	**0.893**
	Lasso_1,1_+LR	0.893	0.981	0.870		0.903	0.853
	Lasso_2,1_+SVM	0.981	0.980	0.886		0.941	0.840
	Lasso_2,1_+LR	0.975	0.973	0.868		0.913	0.847
	Ours+SVM	**0.993**	**0.992**	**0.920**		**0.969**	0.847
	Ours	0.987	0.984	0.895		0.954	0.838

Bold values indicate the largest metrics indifferent models in the same sequence task.

In the single-task experiment, the LR_1_+ SVM model based on the T2 sequence achieved the highest average AUC of 0.973 on the training set, and the average AUC also reached the highest 0.969 on the test set, and accuracy and recall also reached the highest in the single-task model, accuracy = 0.876, recall = 0.886. In all single-task experiments, the maximum value of precision is 0.922.

The multi-task learning model was introduced into the classification of brain tumors, and the classification performance of each model was significantly improved. As can be seen from the data in the table, the values ​​of the indicators of the multi-task model are significantly better than those of the corresponding single-task model. Using our model, the multi-task: Ours+SVM model, its average AUC, accuracy, and precision are all at their highest, and the AUC on the test set reaches 0.992. Our model is not only used as a feature selection method, but also as a classification method. Although its metrics are not optimal, it outperforms the traditional diagnostic models (Lasso_1,1_+SVM, Lasso_1,1_+LR, Lasso_2,1_+SVM, Lasso_2,1_+LR), and we use only one model, thus improving the efficiency of diagnosis.

## 4 Discussion

Accurate classification of GBM and SBM is a challenging clinical problem. Different sequences of MRI provide unique information, and the rational fusion of multiple sequences can complement information from different dimensions (32). Thus, we proposed a new multi-task learning model to enable an accurate and fast diagnosis method for clinical usage.

This study introduced T1, T1C, and T2 sequences into the multi-task learning model. The feature weights were represented as the sum of shared and private weights. In turn, when filtering radiomic features, we can fully use the correlation between MR images of different sequences while still retaining the differences between the sequences and selecting features that have an essential impact on a specific task. Based on the above multi-task model, we also replaced the data matching term with a logistic regression function, which resulted in efficient model feature selection and classification of brain tumors.

We used an alternating minimization algorithm and a fast-iterative shrinkage threshold algorithm to train the data in model solving. We used the 5-fold cross-validation method to select optimal parameters for the selection of parameters. To ensure the accuracy and credibility of the data results, we conducted a 5-fold cross-validation 10 times in training and testing, and the final metrics were selected as the average value. The optimum model with an average AUC of 0.992 on the test set was found when our model performed feature selection and the SVM method performed classification. As a feature selection and classification method, our method alone reached the second highest average AUC of 0.984 on the test set. Multi-task learning enhances the robustness of our model, thus providing a stable predictive model for clinical diagnosis while ensuring accuracy and making diagnoses possible with just one model, improving the efficiency of diagnostics.

Our model still has a comparative advantage over a single sequence classification task. For example, in a previous study (21), we used the same dataset with the random forest method as the feature selection method. Then six machine learning models were used for classification. The final result is that the random forest did the best classification job, obtaining an AUC of 0.97 on the test set but was limited to the T1C sequences. The single-task model, which does not consider the relationship between different sequences, does not take advantage of complementarity of information, which leads to the final classification effect being relatively not very good. On the other hand, the model proposed in this paper can make full use of the complementary information between different sequences and improve the accuracy and robustness of the prediction model. Our model is comparable with the classical multi-task model based on $\ell_{2,1}$ regularization (24), but it can extract not only the same features for each sequence but also features that are important for a specific task. Moreover, compared with the general feature selection model, our model also integrates feature selection and classification to improve efficiency and convenience for diagnosis.

The present study does have some limitations. First, this study used a manual method to segment ROI, which is time consuming. Additionally, although two to three researchers have been involved in the segmentation process, it is very challenging to eliminate all bias in the results. Second, the data sample is small and cannot be extrapolated from this particular population to the general population. Third, the multitask learning model proposed in the present study requires the same features among tasks and does not apply to all multitask problems. Lastly, in the medical imaging part, ROI segmentation requires two neurologists with 5 to 10 years of experience. In our future study, we plan to use deep learning algorithms or image segmentation methods to automatically delineate the ROI to improve the efficiency of our work.

## 5 Conclusion

In this work, we proposed a feature selection model based on the multi-task learning model for SBM and GBM classification. The feature selection model uses a logistic regression function as a loss function, which makes the classification function of the model possible. Most of the current brain tumor classification studies have been performed using single-task models, which do not take advantage of the correlation between different sequences of MR images and therefore, the performance is not optimized to utilize all available information. Furthermore, the traditional multi-task Lasso model does not fully consider the correlation between different tasks. In contrast, our model makes full use of the correlation between MR images of different sequences while selecting the features that have an essential impact on a specific task. It is possible to select different combinations of features for different tasks, thus improving the classification performance of the model to some extent. In conclusion, our model generally outperforms the traditional multi-task Lasso model.

Our model as a feature selection method and paired with an SVM classification method has a great advantage over other methods of the same type. Our proposed model is also a good choice as a classification method. Although it has inferior performance to that of using our method with other classification algorithms, it improves the convenience of tumor classification. Thus, our model is advantageous in classifying SBM and GBM using MR images with multiple sequences.

## Data availability statement

The raw data supporting the conclusions of this article will be made available by the authors, without undue reservation.

## Ethics statement

Studies involving human subjects were reviewed and approved by the ethics committees of Xiangya Hospital, Yunnan Cancer Hospital, and Southern Cancer Hospital. The requirement for informed consent was waived.

## Author contributions

YH: Writing and Data Analysis. SH: Data Analysis. ZL: Founding acquisition; Writing review and editing. All authors contributed to the article and approved the submitted version.

## Funding

The work is supported by Natural Science Foundation of Hunan Province of China with the grant NO.2022JJ30944.

## Acknowledgments

We thank the High-Performance Computing Center of Central South University for providing the computation resource.

## Conflict of interest

The authors declare that the research was conducted in the absence of any commercial or financial relationships that could be construed as a potential conflict of interest.

## Publisher's note

All claims expressed in this article are solely those of the authors and do not necessarily represent those of their affiliated organizations, or those of the publisher, the editors and the reviewers. Any product that may be evaluated in this article, or claim that may be made by its manufacturer, is not guaranteed or endorsed by the publisher.
